# Targeting αvβ3 and αvβ5 integrins inhibits pulmonary metastasis in an intratibial xenograft osteosarcoma mouse model

**DOI:** 10.18632/oncotarget.10461

**Published:** 2016-07-07

**Authors:** Ana Gvozdenovic, Aleksandar Boro, Daniela Meier, Beata Bode-Lesniewska, Walter Born, Roman Muff, Bruno Fuchs

**Affiliations:** ^1^ Laboratory for Orthopedic Research, Department of Orthopedics, Balgrist University Hospital, Zurich, Switzerland; ^2^ Department of Pathology, Institute for Surgical Pathology, University Hospital Zurich, Zurich, Switzerland

**Keywords:** integrins, metastasis, osteosarcoma, targeted therapy, cilengitide

## Abstract

Osteosarcoma is an aggressive bone cancer that has a high propensity for metastasis to the lungs. Patients with metastatic disease face a very poor prognosis. Therefore, novel therapeutics, efficiently suppressing the metastatic process, are urgently needed. Integrins play a pivotal role in tumor cell adhesion, motility and metastasis. Here, we evaluated αvβ3 and αvβ5 integrin inhibition with cilengitide as a novel metastasis-suppressive therapeutic approach in osteosarcoma. Immunohistochemical analysis of αvβ3 and αvβ5 integrins expression in a tissue microarray of tumor specimens collected from osteosarcoma patients revealed that αvβ5 integrin is mainly found on tumor cells, whereas αvβ3 is predominantly expressed by stromal cells. *In vitro* functional assays demonstrated that cilengitide dose-dependently inhibited *de novo* adhesion, provoked detachment and inhibited migration of osteosarcoma cell lines. Cilengitide induced a decline in cell viability, blocked the cell cycle in the G1 phase and caused anoikis by activation of the Hippo pathway. In a xenograft orthotopic mouse model cilengitide minimally affected intratibial primary tumor growth but, importantly, suppressed pulmonary metastasis. The data demonstrate that targeting αvβ3 and αvβ5 integrins in osteosarcoma should be considered as a novel therapeutic option for patients with metastatic disease.

## INTRODUCTION

Osteosarcoma, a highly aggressive bone cancer mainly affecting children and young adults, frequently metastasizes to the lungs [1]. Metastases are detectable in 20% of the patients at the time of diagnosis and 80% of the patients initially presenting with localized disease develop metastases later on [2]. Patients with metastases have a long-term survival rate of only 20% despite multiple efforts to improve treatment efficacies [3]. Therefore, the development of innovative therapeutic strategies targeting metastasis, the major cause of death of osteosarcoma patients, is urgently needed to improve the prognosis of these patients.

Integrins, a family of heterodimeric cell surface receptors consisting of α and β subunits, are well-known adhesion molecules expressed in both tumor cells and tumor-associated healthy cells [4]. Through binding to various extracellular matrix proteins, integrins affect tumor cell attachment, survival, motility, invasion and metastasis. Preclinical and clinical research on integrins in cancer, particularly on αvβ3 and αvβ5, revealed their substantial potential as therapeutic targets for the treatment of different cancer types [5, 6]. Cilengitide, a cyclic RGD-containing pentapeptide, was recognized as a selective inhibitor of αvβ3 and αvβ5 integrin-mediated cell-cell and cell-matrix interaction that exhibited strong anti-angiogenic, anti-tumor, anti-invasive activity and anti-metastatic activity in various animal models of cancer [7–13]. Furthermore, cilengitide displayed a favorable safety profile and entered clinical trials for treatment of multiple cancer types [14, 15].

At present, little is known about the contribution of αvβ3 and αvβ5 integrins to osteosarcoma progression and metastasis. αvβ3 integrin expression was found to correlate with the migratory and chemotactic activity of human osteosarcoma cells towards lung tissue homogenates as indicators of metastatic potential [16]. Depletion of αv integrins in human SaOS-2 osteosarcoma cells through expression of an intracellular antibody suppressed MMP-2 expression and the induction of bone differentiation markers, implying that αv integrin regulates the osteosarcoma cell phenotype [17]. The expression of αvβ3 and αvβ5 heterodimers in human osteosarcoma tissue has so far not been investigated.

Here, we investigated αvβ3 and αvβ5 integrins as putative therapeutic targets in osteosarcoma. We demonstrate that both integrins are present in tumor specimens collected from osteosarcoma patients and in established cell lines. Inhibiting their function with cilengitide suppressed adhesive and migratory properties of osteosarcoma cells *in vitro*, decreased the viability and lead to activation of the Hippo signaling pathway. A preclinical therapy study in a metastasizing intratibial osteosarcoma mouse model showed that intraperitoneal treatment of the mice with cilengitide diminished pulmonary metastasis. Our findings highlight αvβ3 and αvβ5 integrins as attractive targets for metastasis suppressive treatment in osteosarcoma which deserves further investigation.

## RESULTS

### αvβ3 and αvβ5 integrins are expressed in tumor tissue of osteosarcoma patients and in established osteosarcoma cell lines

**Figure 1:**
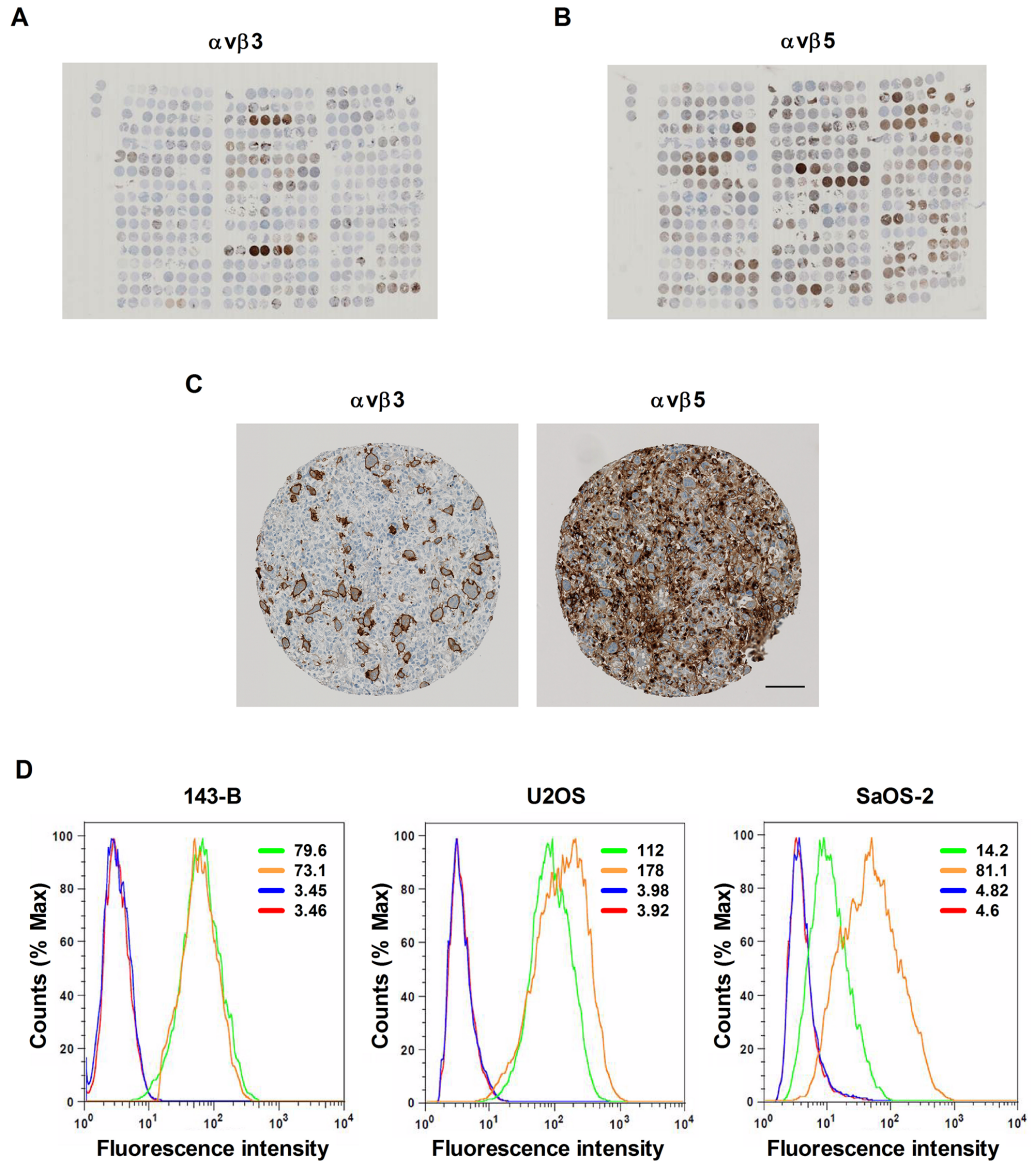
Expression of αvβ3 and αvβ5 integrins in human osteosarcoma tissue arranged on a tissue microarray and in established human osteosarcoma cell lines **A.** Overview images of αvβ3 - or **B.** of αvβ5 immunostaining of 4.5 μm tissue microarray sections consisting of primary biopsies, surgical resections, lung and bone metastases and biopsies of recurrent disease derived from osteosarcoma patients. **C.** Immunohistochemical staining of αvβ3 and αvβ5 integrins in a representative TMA spot. Scale bar, 100 μm. **D.** αvβ3 and αvβ5 integrin expression in indicated osteosarcoma cell lines was determined by flow cytometry and expressed as mean fluorescence intensity (values); green: αvβ3; orange: αvβ5; blue: secondary antibody control; red: non-stained control.

The expression of αvβ3 and αvβ5 integrins was investigated in tumor specimens collected from osteosarcoma patients and arranged in a tissue microarray. Representative tissue microarray sections stained with antibodies to αvβ3 and αvβ5 integrins are presented in Figure 1A and 1B, respectively. Immunostained αvβ3 and αvβ5 integrins displayed very different distribution patterns in individual tissue microarray spots (Figure 1C). The results of the immunohistochemical evaluation of αvβ3 and αvβ5 staining pattern, intensity and grading score are presented for each tissue microarray core in Supplementary Table S1. αvβ3 was predominantly found in stromal cells of osteosarcoma biopsies, resections, metastases and recurrences, whereas αvβ5 integrin was predominantly observed on the majority of tumor cells. Interestingly, αvβ3 was expressed on the stromal cells in 38 out of 64 biopsies, whereas αvβ3 immunostained tumor cells were only found in 1 out of the 64 biopsies. In contrast, αvβ5 was detected on tumor cells in 51 out of 60 biopsies. Likewise, a very high percentage of metastases and recurrences were positive for both integrins. In both tumor and stromal cells the two integrins were mainly localized in membrane structures. Stromal cells stained with αvβ3 specific antibodies included multinucleated giant osteoclast-like cells. αvβ3 and αvβ5 immunostaining was not observed in the tumor vasculature. Moreover, the expression of αvβ3 and αvβ5 in tumor tissue did not correlate with clinico-pathological characteristics or the survival of the patients (data not shown).

A parallel analysis of the expression of αvβ3 and αvβ5 integrins at the cell surface of osteosarcoma cell lines by flow cytometry showed that both heterodimers were expressed by all the cell lines examined (Figure 1D). However, the mean fluorescence intensities indicated that the expression varied among the cell lines. SaOS-2 cells expressed the lowest amounts of αvβ3, whereas the highest amounts of αvβ5 integrin were observed in U2OS cells.

In summary, αvβ3 and αvβ5 integrins were found expressed in the majority of the human osteosarcoma tissue specimens as well as in all osteosarcoma cell lines investigated in the present study.

### Cilengitide dose-dependently inhibits *de novo* adhesion to vitronectin, causes detachment and impairs migration of osteosarcoma cells

**Figure 2:**
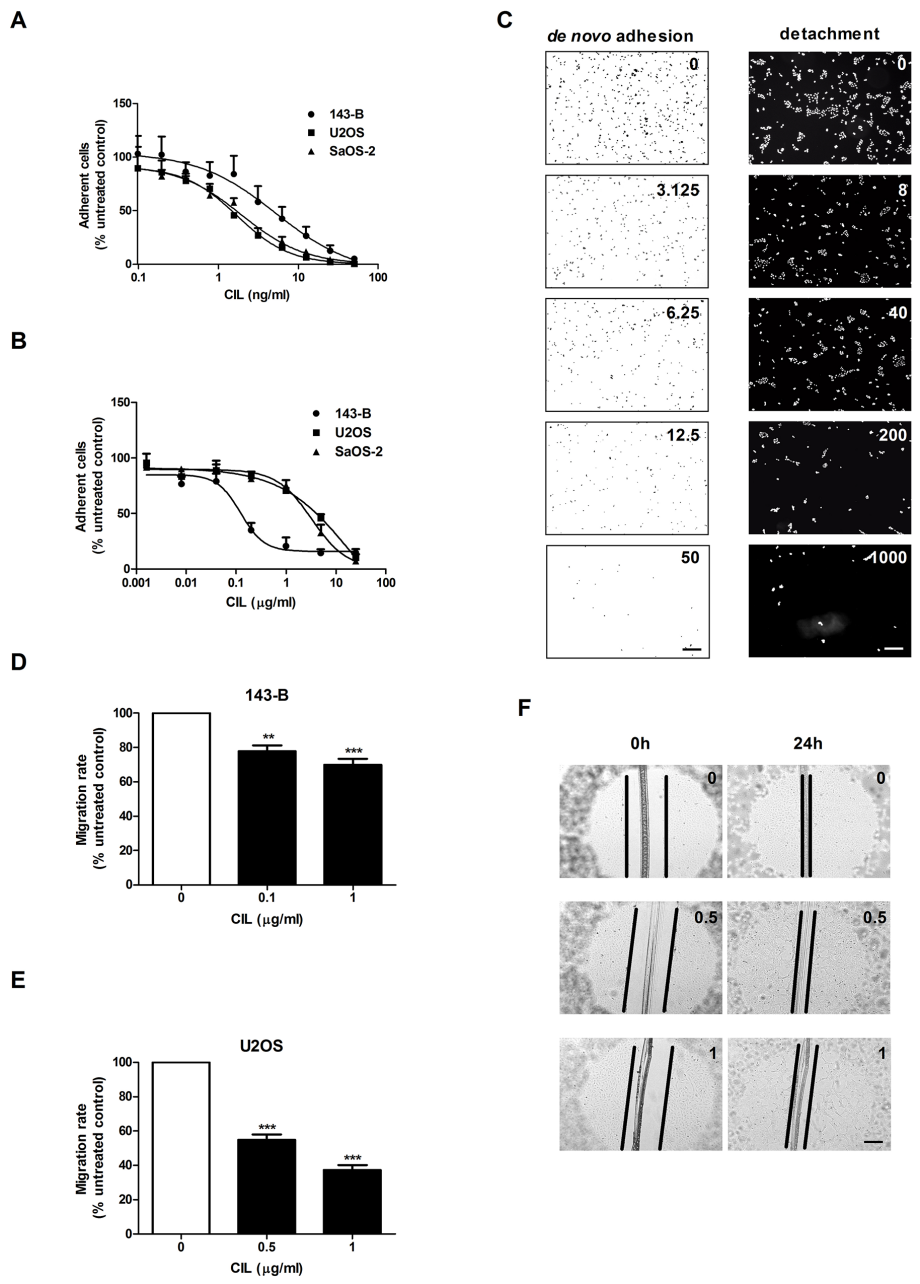
Cilengitide (CIL) inhibits *de novo* adhesion and causes detachment of osteosarcoma cells from vitronectin and reduces cell migration **A.** CIL dose-dependent inhibition of *de novo* adhesion (15 minutes) of indicated osteosarcoma cell lines. **B.** CIL dose-dependent detachment (2 hours) of indicated pre-attached osteosarcoma cells. **C.** Representative images of adherent 143-B cells in the absence or presence of indicated concentrations (ng/ml) of CIL. Cell nuclei were visualized by crystal violet staining in the *de novo* adhesion assay (left panels) and by DAPI staining in the detachment assay (right panels). Scale bars, 250 μm. **D.** Effects of cilengitide on the migration of 143-B and **E.** of U2OS-2 cells. **F.** Representative images of wounds in U2OS cell monolayers examined microscopically immediately after wounding and after 24 hours of incubation with CIL at indicated concentrations (μg/ml). Scale bar, 250 μm. Values in A, B, D and E are expressed as the mean ± SEM of at least 3 independent experiments; **, *P* < 0.01; ***, *P* < 0.001 vs non-treated control.

Integrins are known to be involved in adhesion and migration processes during the metastatic progression and vitronectin is an extracellular matrix component binding to both αvβ3 and αvβ5 integrins. Cilengitide dose-dependently inhibited *de novo* adhesion of single 143-B, U2OS and SaOS-2 cells to vitronectin (Figure 2A). Moreover, cilengitide also detached 143-B, U2OS and SaOS-2 cells in sub-confluent monolayers grown on vitronectin already after 2 hours of treatment in a dose dependent manner (Figure 2B). Representative images of 143-B cells adherent to vitronectin in *de novo* adhesion and detachment assays in the absence or presence of indicated cilengitide concentrations are shown in Figure 2C. Interestingly, the data illustrate that approximately 1000-times higher concentrations of cilengitide are needed to detach the here investigated osteosarcoma cell lines from vitronectin than to inhibit their *de novo* adhesion to vitronectin. Remarkable differences in *de novo* adhesion of the cell lines to non-coated or vitronectin-coated culture dishes in serum-free medium confirmed that vitronectin promotes adhesion and that cilengitide interferes with this process (Table 1). Detachment experiments carried out in serum-containing medium showed that cilengitide detached the osteosarcoma cells at comparable concentrations from non-coated or vitronectin-coated plastic. This indicated that vitronectin of serum origin provided sufficient plastic coating in these experiments as previously reported [8, 18]. Consequently, all subsequent experiments with fully attached cells were performed without previous vitronectin coating.

**Table 1:** Effects of cilengitide on cell adhesion

Cell line	*De novo* adhesion (serum-free media)	Detachment (serum-containing media)	*De novo* adhesion (serum-free media)	Detachment (serum-containing media)
	with Vitronectin coating		without Vitronectin coating	
	IC50 (ng/ml)			
**143-B**	5.2	100	n.a.^a^	200
**U2OS**	1.7	5600	n.a.	9300
**SaOS-2**	1.9	3800	n.a.	3800

a n.a.(not applicable); *De novo* adhesion too low in the absence of vitronectin

The impact of cilengitide on the migration activity of osteosarcoma cell lines was assessed in a wound healing assay using confluent cells, which showed that the migration rates were dose-dependently reduced by cilengitide. As shown in Figure 2D, the migration of 143-B cells treated with 0.1 or 1 μg/ml of cilengitide was decreased by 22 ± 3.3% or 30 ± 3.6%, respectively, compared to that of non-treated cells (*P* < 0.01). Interestingly, cilengitide had a more pronounced effect on the migration of U2OS cells. There 0.5 and 1 μg/ml cilengitide reduced the migration by 46 ± 3.1% and 62.7 ± 3.3%, respectively, compared to non-treated controls (Figure 2E; *P* < 0.001). Cilengitide-treated cells remained fully attached, but displayed a partial loss of intercellular contacts as presented in Figure 2F. It has been previously reported that effects of cilengitide depend on the cell confluency [19]. Accordingly, in our experiments, the cell confluency and the duration of treatment influenced cilengitide efficacy in *in vitro* functional assays. Along these lines, the migration of SaOS-2 cells could not be evaluated in this assay, because cilengitide treatment of confluent cells for 24 hours resulted in considerable detachment of cells (data not shown).

Taken together, the data presented here demonstrate that cilengitide inhibits *in vitro* metastatic properties of the osteosarcoma cells investigated.

### Cilengitide decreases cell viability and induces G1-cell cycle arrest *in vitro*

**Figure 3:**
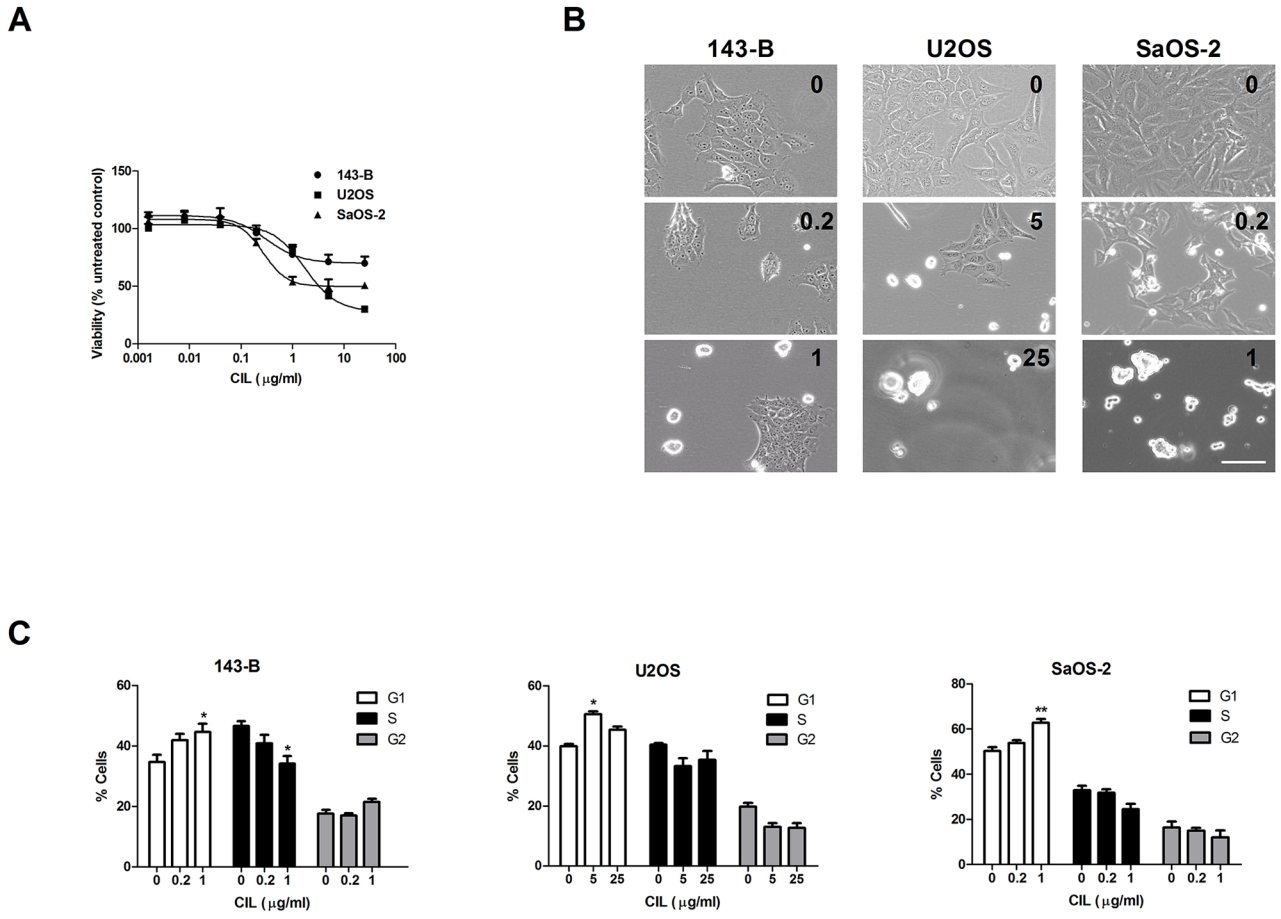
Effects of cilengitide (CIL) on cell viability, morphology and cell cycle progression **A.** Viability of indicated cell lines assessed in a WST-1 assay after incubation for 24 hours with CIL at indicated concentrations. **B.** Representative phase-contrast micrographs of cells treated with indicated concentrations (μg/ml) of CIL for 24 hours (143-B, U2OS) or 48 hours (SaOS-2) prior to cell cycle analysis. Scale bar, 100 μm. **C.** Cell cycle distribution (% of total cell population) of indicated osteosarcoma cell lines, assessed by flow cytometric analysis of propidium iodide-stained cells, after treatment for 24 hours (143-B, U2OS) or for 48 hours (SaOS-2) with CIL at indicated concentrations. Values in A and C represent the mean ± SEM of at least 3 independent experiments; *, *P* < 0.05; **, *P* < 0.01 vs untreated control.

The treatment of 143-B cells with increasing concentrations of cilengitide for 24 hours had only a modest effect on their viability, reflected by a maximal decline of only 30% assessed in a WST-1 assay (Figure 3A). U2OS and SaOS-2 cells, on the other hand, showed a higher sensitivity, which resulted in a loss of viability of 70% and 49%, respectively (Figure 3A). Extending the treatment period to 72 hours did not lead to greater maximal effects of cilengitide (data not shown). However, cilengitide incubation provoked striking morphological changes and ultimately cell detachment (Figure 3B).

We hypothesized that the limited impact of cilengitide on cell viability is associated with changes in cell-cycle progression. Indeed, a flow cytometric analysis of cell-cycle progression upon cilengitide treatment indicated a G1 cell-cycle arrest in all three cell lines investigated (Figure 3C). The G1-phase arrest in cilengitide-treated cells was accompanied by lower cyclin D1 expression than that observed in non-treated cells (Supplementary Figure S1).

We subsequently investigated whether cilengitide provokes apoptotic cell death in osteosarcoma cell lines by Western blot analysis of PARP cleavage. Minimal or no PARP cleavage was observed in 143-B and SaOS-2 cells (data not shown). In contrast, U2OS cells responded to 24 hour cilengitide treatment with a dose-dependent increase in PARP cleavage (Supplementary Figure S2A). Interestingly, the observed PARP cleavage was not inhibited by Z-VAD, indicating caspase-independent apoptosis. This finding was consistent with no change in viability of cells treated with cilengitide in the absence or presence of Z-VAD (Supplementary Figure S2B).

Collectively, these data show that cilengitide has a moderate impact on cell viability and provokes G1-phase cell cycle arrest. Cilengitide exhibited a cytostatic effect in the 143-B and SaOS-2 cell lines. In U2OS cells, on the other hand, the cilengitide-evoked cell cycle arrest was followed by caspase-independent apoptosis.

### Targeting of αvβ3 and αvβ5 integrin with cilengitide *in vitro* activates the Hippo pathway

**Figure 4:**
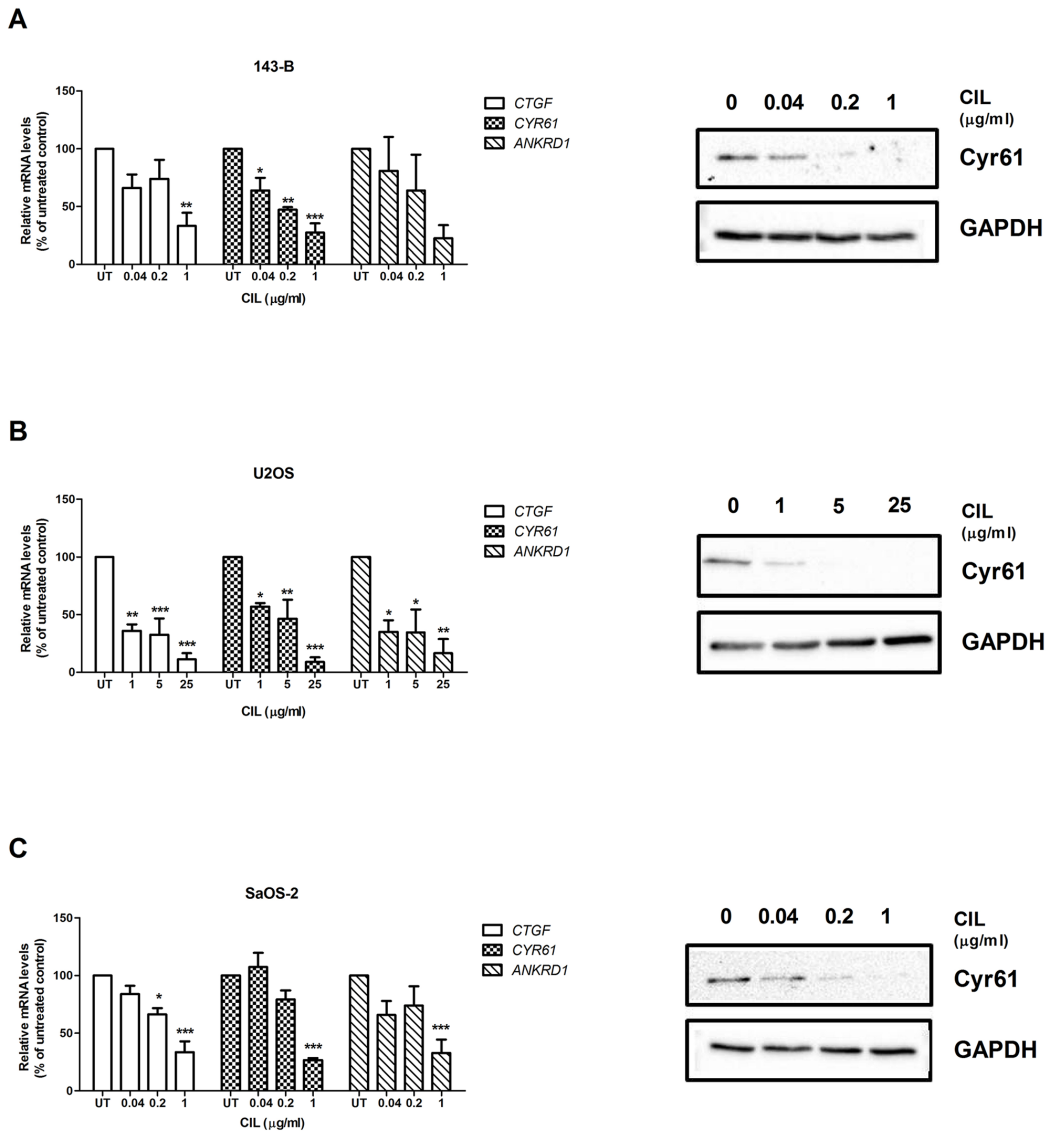
Cilengitide (CIL) activates the Hippo pathway The levels of mRNA encoding the YAP target genes CTGF, CYR61 or ANKRD1 were analyzed by quantitative RT-PCR (left panels) and normalized to those of GAPDH in total RNA extracts of 143-B **A.** U2OS **B.** or SaOS-2 cells **C.** left untreated or treated with indicated concentration of CIL for 24, 6 and 6 hours, respectively. mRNA levels normalized to those of GAPDH in non-treated control cells were set to 100%. Values are presented as the mean ± SEM of at least 3 independent experiments; *, *P* < 0.05; **, *P* < 0.01; ***, *P* < 0.001 vs non-treated control. The expression of CYR61 at the protein level and of GAPDH as a reference (right panels) was analyzed on Western blots of whole cell extracts of the indicated cell lines treated for 24 hours with CIL at indicated concentrations. Representative Western blots of three independent experiments.

Recently, it has been reported that cell detachment activates the tumor suppressor Hippo pathway, leading to inhibition of YAP transcriptional co-activator and to anoikis [20]. We, therefore, speculated that targeting αvβ3 and αvβ5 integrins with cilengitide might activate the Hippo pathway. This hypothesis was tested by examining the expression of well characterized YAP target genes in osteosarcoma cell lines treated with cilengitide for indicated time periods. In order to exclude an impact of cell viability on gene expression, U2OS and SaOS-2 cells were treated for only 6 hours, a time period within which cilengitide did not have a significant effect on cell viability (Supplementary Figure S2C and S2D). Cilengitide indeed inhibited dose-dependently the expression of mRNA from the three YAP target genes CTGF, CYR61 and ANKRD1 (Figure 4A, 4B, and 4C, left panels). The downregulation of CYR61 was furthermore confirmed at the protein level in all cell lines (Figure 4A, 4B, 4C, right panels).

The results indicate that αvβ3 and αvβ5 integrins might be upstream regulators of the Hippo signaling pathway.

### Cilengitide treatment minimally affects intratibial primary tumor growth, but suppresses pulmonary metastasis in a xenograft osteosarcoma mouse model

**Figure 5:**
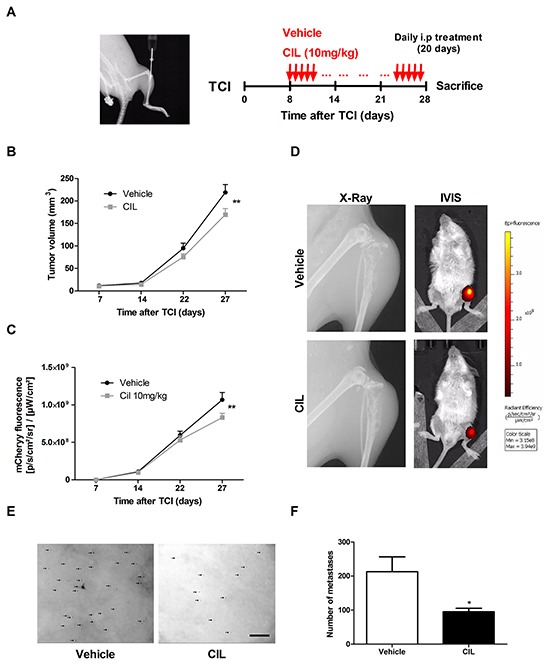
Cilengitide (CIL) inhibits pulmonary metastasis *in vivo* **A.** X-Ray image of the site of intratibial injection of 143-B/mCherry/LacZ cells taken at the time of the tumor cell injection (TCI) on Day 0 (left panel). Protocol for the treatment of tumor bearing mice with vehicle (control) or CIL (10 mg/kg body weight) (right panel). **B.** Primary tumor growth over time monitored by caliper measurements of the tumor volume at indicated time points in mice treated with vehicle or with CIL. **C.** mCherry fluorescence indicating primary tumor growth over time in the same vehicle- or CIL-treated animals. **D.** X-Ray images of tumor bearing legs and IVIS images of tumor bearing animals taken at the end of the experiment from representative mice treated with vehicle or with CIL. **E.** Representative pictures and **F.** quantification of X-gal stained pulmonary metastases (arrows) on lung mounts collected and prepared as described in the *Materials and Methods* from vehicle or CIL treated mice at sacrifice 28 days after TCI. Scale bar, 250 μm. Values in B, C and F are the mean ± SEM of the data collected in 12 vehicle- and 11 CIL-treated mice; *, *P* < 0.05; **, *P* < 0.01.

*In vivo* tumor- and metastasis suppressive activity of cilengitide was investigated in a well-established 143-B/mCherry/LacZ cell line-derived intratibial osteosarcoma model in SCID mice that mimics the human metastatic disease with osteolytic bone lesions and lung metastases (Figure 5A, left panel) [21]. Daily intraperitoneal administration of cilengitide (10 mg/kg body weight) or vehicle for 20 days was started 8 days after intratibial tumor cell inoculation (Figure 5A, right panel). Periodic assessment of primary tumor size by caliper measurements and by mCherry fluorescent imaging showed only a minor inhibitory effect of cilengitide on primary tumor growth (Figure 5B and 5C). A significantly (*P* < 0.01) smaller mean primary tumor volume in cilengitide-treated mice than in vehicle-treated animals was only observed at the end of the therapy on day 27 after tumor cell inoculation. Respective mean final tumor volumes assessed by caliper measurements amounted to 169 ± 14 mm^3^ and 219 ± 17 mm^3^, respectively. Consistent with these results, mCherry fluorescent signals detected in the tumor-bearing legs of living animals were significantly (*P* < 0.01) lower in cilengitide-treated mice than in vehicle-treated control mice at the end of the therapy (Figure 5C). X-Ray and IVIS images, presented in Figure 5D, show osteolysis and mCherry fluorescence in proximal tibias of representative mice treated with vehicle or cilengitide for 20 days. Importantly, cilengitide treatment had a much more pronounced inhibitory effect on pulmonary metastasis, assessed *ex-vivo* in X-Gal stained lungs, than on primary tumor growth (Figure 5E). The mean number of metastatic foci was 2.2 times lower in lungs of cilengitide-treated mice than in lungs dissected from vehicle-treated animals (Figure 5F, *P* < 0.05).

Taken together, cilengitide, selectively targeting αvβ3 and αvβ5 integrins, has a limited impact on primary tumor development in the intratibial xenograft osteosarcoma mouse model investigated here. However, it significantly inhibited lung metastasis demonstrating that αvβ3 and αvβ5 integrin targeting restrains metastatic spread of osteosarcoma.

## DISCUSSION

Osteosarcoma is the second leading cause of cancer-associated death in childhood and adolescence due to its high potential for metastatic spread [22]. The identification of metastasis promoting mechanisms followed by the development of new therapeutic compounds that efficiently inhibit these mechanisms will finally help to reduce the currently high mortality rate of osteosarcoma patients with metastatic disease.

The present study shows for the first time that αvβ3 and αvβ5 integrins are expressed in the majority of tumors of osteosarcoma patients and that selective targeting of these integrins needs to be considered as novel metastasis-suppressive treatment approach in osteosarcoma. Interestingly, αvβ5 integrin was found to be predominantly expressed by tumor cells whereas αvβ3 integrin was mainly recognized on stromal multinucleated osteoclast-like cells. These intratumoral benign cells have been observed in 25% of osteosarcomas [23]. Our findings are consistent with those of a report demonstrating that αvβ3 integrin surface expression is essential to the formation of osteoclast-like multinucleated cells [24]. Our observations confirm previously reported high expression of all αv integrins in tumor specimens collected from twenty osteosarcoma patients [25]. The expression pattern of αvβ3 integrin in osteosarcoma is similar to that described in breast and gastric cancer [26, 27]. Furthermore, abundant expression of integrins described for brain metastases from various primary malignancies is in good agreement with our findings [26, 28]. However, unlike several recent studies in a variety of human cancers employing the integrin antibodies used in our study, we did not observe immunoreactivity in tumor vascular structures [28, 29]. As integrins investigated here are expressed in both the tumor cells and the stromal components of osteosarcoma, their targeting with cilengitide might be a particularly effective treatment approach since it is simultaneously directed towards the tumor cells and the tumor microenvironment.

The here reported results of an *in vivo* study, carried out in an orthotopic xenograft mouse model that closely mimics the human disease, demonstrate a predominant anti-metastatic activity of cilengitide, implicating that αvβ3/αvβ5 integrins play an important role during metastasis of osteosarcoma. These results are in line with those of the *in vitro* experiments, demonstrating that cilengitide suppressed cellular adhesion and motility, important properties of metastasizing cells, in a panel of αvβ3/αvβ5 expressing osteosarcoma cell lines much like in meningioma [30]. Using clinically relevant concentrations of cilengitide *in vitro,* we observed inhibition of *de novo* adhesion to and detachment from vitronectin, well-known effects of cilengitide observed in a variety of cancer cell lines [8, 19, 31]. Cell-cell interactions between cancer cells and endothelium were also found to be crucial in the metastatic process. It was shown that αvβ3 integrin on melanoma cells interacts with Thy1 on endothelial cells in order to facilitate metastasis [32]. Therefore, in addition to the here reported inhibition of adhesion to the extracellular matrix by cilengitide, it is likely that the compound also inhibited the interaction of osteosarcoma cells with the vasculature and thereby further contributed to the *in vivo* observed suppression of metastasis. Different from findings in breast carcinoma and multiple myeloma [13, 33], in our osteosarcoma model cilengitide did not inhibit osteolysis, examined by micro-computed tomography (data not shown), implying that, under our treatment settings, bone resorption by osteoclasts was not suppressed. The modest anti-tumor effect of cilengitide seen *in vivo* is in line with the *in vitro* the G1-cell cycle block, recognized for the first time in our study, and induction of anoikis resulting in a limited effect on viability. In melanoma, meningioma and breast carcinoma animal models, much like in our model, cilengitide, used as a single drug, did not affect tumor growth, but a synergistic effect was observed when cilengitide treatment was combined with chemotherapy or radiotherapy [30, 34, 35]. Thus, such a combination therapy needs to be envisaged in future osteosarcoma treatment studies.

In order to investigate alterations in cell signaling upon inhibition of integrins we focused on the Hippo signaling pathway, an evolutionarily conserved tumor suppressor pathway [36]. Activation of the Hippo pathway leads to degradation of the YAP protein, thereby inhibiting its growth promoting function. Dysregulation of this pathway contributes to various aspects of cancer progression, including metastasis. Hippo pathway inactivation has been observed in a variety of human cancers [37]. Abundant expression of YAP has also been found in osteosarcoma [38]. In the recent years, the Hippo pathway received substantial attention and key components and several negative and positive regulators have been characterized [39]. However, cell surface-located regulatory components of the Hippo pathway are largely unknown. Here, the αvβ3 and αvβ5 integrins were for the first time identified as negative regulators of the Hippo pathway in osteosarcoma cells. This was reflected by the suppression of YAP target genes including the CYR61 gene upon integrin inhibition. Interestingly, CYR61, found downregulated in cilengitide-treated cells, has been recognized as a metastasis promoting protein in osteosarcoma [40]. Our data are in line with recent findings demonstrating that inhibition of integrin-linked kinase activity in breast, prostate and colon cancer cells activated the Hippo pathway [41].

Unfortunately, cilengitide failed to improve the survival of glioblastoma patients in a Phase III trial, and its development as an anti-cancer drug was subsequently cancelled [15]. Nevertheless, an integrin-targeting therapeutic strategy remains an attractive option in cancer management.

In conclusion, αvβ3 and αvβ5 integrin targeting, reactivating the Hippo signaling pathway, has potential as a strategy for metastasis suppressive treatment in osteosarcoma and presumably in other malignancies and should be further explored in clinical settings.

## MATERIALS AND METHODS

### Human osteosarcoma tissue microarray analysis

Osteosarcoma tissue specimens, including biopsies and primary tumor, relapse and metastases surgical resections, were collected between June 1990 and December 2005 from 86 patients in accordance with the regulations of the local ethic committee. Clinical data of the patients are presented in Supplementary Table S2. A tissue microarray was constructed as described [42]. The immunostaining for αvβ3 and αvβ5 integrins was performed with anti- αvβ3 (clone EM22703) and anti-αvβ5 (clone EM09902) rabbit monoclonal antibodies kindly provided by Simon L. Goodman as recently reported [26]. The immunohistochemical evaluation of αvβ3 integrin expression was carried out individually for tumor and stromal cells and the absence (negative) or presence (positive) of immunoreactive staining was judged by eye. Grading of αvβ5 integrin immunohistochemical staining, based on the intensity and the percentage of immunostained area, was done with a custom made MATLAB (v2009b, Mathworks Inc) program as described [43].

### Cell culture and reagents

The human osteosarcoma cell lines 143-B (CRL-8303), U2OS (HTB-96) and SaOS-2 (HTB-85) were obtained from American Type Culture Collection (ATCC). Cells were stably transduced with a LacZ gene, selected as described [43–45], and cultured in DMEM (4.5 g/l glucose)/HamF12 (1:1) medium (Invitrogen) supplemented with 10% heat inactivated fetal bovine serum (FBS, Gibco) (referred to as tissue culture medium) at 37°C in a humidified atmosphere of 5% CO_2_ and 95% air. The cell lines were authenticated by short tandem repeat DNA profiling (Microsynth) with a PowerPlex®16HS system (Promega) and by comparison with the German Collection of Microorganisms and Cell Cultures database (DSMZ). Cilengitide (EMD121974) was provided by Merck KgaA. Cisplatin was purchased from Sigma-Aldrich. Z-VAD-FMK (referred to in the text as Z-VAD) was purchased from BD Pharmingen (550377).

### Analysis of integrin expression in osteosarcoma cell lines

Cells were detached in a buffer for flow cytometry (1 × PBS; 4 mM EDTA; 2% FBS) and incubated on ice with antibodies to αvβ3 (clone LM609, MAB1976, Millipore, dilution 1:200) and αvβ5 (clone P1F6, MAB1961, Millipore, dilution 1:200) integrins for 30 minutes. After washing twice with the buffer described above, the cells were further incubated on ice in the dark with a fluorescently-conjugated secondary antibody (IgG-PE, Novus Biologicals, 20103, dilution 1:200) for 20 minutes. Non-bound secondary antibodies were removed by additional washing steps. Control cells were not stained or incubated with the secondary antibody alone. The mean fluorescence intensity of the fluorescently-labeled cells was then analyzed on a FACS machine (Calibur, BD) and with FlowJo software (Tree Star).

### Adhesion assay

Adhesion assays were carried out in 96-well plates. The wells were coated with 200 ng/cm^2^ of vitronectin (Sigma-Aldrich, SRP3186) diluted in PBS supplemented with 0.1% BSA for 1 hour at room temperature (RT). After washing with PBS, the wells were blocked with heat-denatured (HD) 1% BSA in PBS for 1 hour at RT. Wells coated with HD-BSA alone were used as controls. In the *de novo* adhesion assay, cells were detached with accutase (Sigma-Aldrich, A6964), resuspended in DMEM/F12/0.1% BSA and 5 × 10^3^ cells in 50 μl were seeded in triplicates into wells containing 50 μl of increasing concentrations of cilengitide and allowed to adhere for 15 minutes at 37°C. In the detachment assay, 2 × 10^3^ cells/well were seeded in triplicates in 80 μl of tissue culture medium into vitronectin-coated wells and left to fully adhere overnight. The next day, 20 μl of cilengitide at increasing concentrations were added to the wells and incubated for 2 hours at 37°C. Non-adherent cells were removed by washing with PBS and adherent cells were fixed with 10% formalin in PBS for 15 minutes at RT and then stained with 0.05% crystal violet in H_2_O or DAPI (1:1000) for 15 minutes at RT in the *de novo* adhesion assay and the detachment assay, respectively. Images of randomly selected areas of 3.6 mm^2^ were taken with an AxioCam MRm camera connected to the Zeiss Observer.Z1 inverted microscope (Carl Zeiss MicroImaging GmbH) set at 4x magnification. The number of adherent cells in the analyzed area was determined with ImageJ software (http://rsb.info.nih.gov/ij/) and the total number of adherent cells per well was then calculated. The number of adherent cells treated with cilengitide was normalized to the number of adherent untreated cells set to 100%. The data of three independent experiments are presented.

### Migration assay

The migratory properties of osteosarcoma cells were assessed in a wound healing migration assay. Cells were seeded into 6-well plates. At confluency, six wounds per well measuring between 0.3 and 1 mm in width and approximately 1 cm in length were applied with a sterile pin. Cell debris was removed by washing twice with tissue culture medium. Tissue culture medium with or without indicated concentrations of cilengitide was then added to the wells and 143-B or U2OS cells were incubated for 7 or 24 hours, respectively. Wounds were marked with a Nikon object marker attached to a Nikon Diaphot microscope. Photos of the marked wounds were taken with an AxioCam MRm camera connected to the Zeiss Observer.Z1 inverted microscope (Carl Zeiss MicroImaging GmbH) set at 4x magnification immediately after wounding and after the incubation with cilengitide. The width of the marked wound areas were measured with the ImageJ software (http://rsb.info.nih.gov/ij/). The migration rate (μm/h) was calculated according to the formula (D_0_-D_t_)/2t, where D_0_ is the wound width immediately after wounding and D_t_ is the wound width after the incubation for the indicated time (t). The migration rate of untreated cells was set to 100%. The experiments were done in duplicates and repeated at least three times.

### Cell viability assay

Cell viability assays were performed in 96-well plates. 2 × 10^3^ 143-B or 5 × 10^3^ U2OS or SaOS-2 cells per well were seeded in tissue culture medium and allowed to adhere overnight. Increasing concentrations of cilengitide were added the next day and the cells were incubated for 6 or 24 hours. Following cilengitide treatment, the cells were incubated with 10 μl/well of WST-1 reagent (Roche, 05015944001) for 3 hours and the cell viability was then assessed as previously described [46]. Three independent experiments in triplicates were performed.

### Cell cycle analysis

Cell cycle progression was measured by propidium iodide (PI) staining using flow cytometry. Briefly, tissue culture medium alone containing different concentrations of cilengitide were added to fully attached and spread cells at approximately 50% confluency and incubated for 24 hours (143B, U2OS) or 48 hours (SaOS-2) according to the doubling time of the respective cell lines. Following the treatment, adherent cells were trypsinized and collected together with the floating cells, washed once with cold PBS and resuspended in 300 μl of PBS. Subsequently, the cells were fixed in ice cold ethanol and stored at −20°C overnight. The next day, DNA was stained in PI/RNase staining buffer (BD Pharmingen, 550825) for 30 minutes at 37°C in the dark. The samples were analyzed on a FACS machine (Calibur, BD) and the cell cycle distribution in % was calculated using FlowJo software (Tree Star). The experiments were performed in triplicates and repeated three times.

### Western blotting

The preparation of protein extracts and Western blot analysis were performed as previously described [43]. Antibodies used were anti-PARP (9542, dilution 1:1000) obtained from Cell Signaling Technology, anti-actin (MAB1501, dilution 1:10000) from Millipore, and anti-GAPDH (FL-335, dilution 1:3000), anti-Cyr61 (sc271217, dilution 1:1000), anti-CyclinD1 (sc20044, dilution 1:1000) and HRP-conjugated secondary antibodies (sc2006, sc2055, dilution 1:5000) were purchased from Santa Cruz Biotechnologies. Peroxidase activity was visualized with Immobilon chemoluminescence substrate (Millipore, WBKLS0500) and a VersaDocTM Imaging System (Bio-Rad).

### RNA extraction, cDNA synthesis and quantitative real-time polymerase chain reaction (qPCR)

Total RNA from cells treated with various concentrations of cilengitide or left untreated was isolated with a RNeasy Mini Kit (Qiagen, 74104) according to the manufacturer's instructions. 1 μg of total RNA was transcribed to cDNA with a High-Capacity cDNA Reverse Transcription Kit with Rnase Inhibitor (Applied Biosystems, 4374966) as described in the protocol provided by the manufacturer. The cDNA was diluted in nuclease-free water and real-time qPCR was conducted on cDNA equivalent to 10 ng of starting RNA with the Power SYBR Green PCR Master Mix (Applied Biosystems, 4367659) on a StepOne-Plus Real-Time PCR System (Applied Biosystems). The denaturation was performed for 10 minutes at 95°C followed by 40 PCR cycles for 15 seconds at 95°C and for 1 minute at 60°C. The analysis was done with StepOne Software version 2.1 (Applied Biosystems). Relative expression levels were calculated by the comparative (ΔΔCT) method and normalized to GAPDH. All primers used are listed in Supplementary Table S3. The experiments were done in triplicates and repeated three times.

### Cilengitide treatment in an intratibial xenograft osteosarcoma model in SCID mice

The animal study was approved by the Ethics Committee of the Veterinary Office of the Canton Zurich and was conducted in accordance with the Swiss Animal Protection Law. In order to enable visualization of tumor cells within mouse tissues *in vivo* and *ex vivo*, 143-B/LacZ cells were transduced with an mCherry gene (143-B/mCherry/LacZ cells) as described recently [21]. Eight to ten week old SCID/CB17 immunocompromised mice purchased from Charles River Laboratories were intratibially injected with 10 μl of 10^5^ 143-B/mCherry/LacZ cells in PBS/0.05% EDTA on day 0. Eight days after tumor cell injection (TCI), the mice were randomly distributed into two groups and daily intraperitoneal injections of 10 mg/kg body weight of cilengitide (treatment group) or vehicle (physiological saline) (control group) were initiated. The cilengitide-treated group consisted of 11 mice, whereas the vehicle-injected control group included 12 mice. Primary tumor development was examined weekly by X-ray with an MX-20 DC Digital Radiography System (Faxitron X-Ray Corporation) and by mCherry fluorescence imaging with an IVIS imaging system (Caliper Life Sciences, Inc.). The mCherry fluorescence was quantified with a Living Image software version 3.1 (Xenogen Corporation). Caliper measurements of the length and the width of the tumor leg were performed weekly and the primary tumor volume was calculated with the formula V=length × width^2^/2. The volume of the non-injected leg was used as a reference value. The animals were sacrificed after 20 days of treatment and the primary tumors and *in situ* perfused lungs were harvested as reported [45]. Organs were fixed for 30 minutes at RT in 2% formaldehyde and X-gal stained as described [47, 48]. Indigo-blue stained pulmonary metastases on the lung surface were counted at 4x magnification under the Nicon Eclipse E600 microscope (Nikon Corporation).

### Statistical analysis

Statistical significance of differences between the experimental groups was determined using a Student t test or ANOVA and *P* < 0.05 was considered significant. All analyses were performed using GraphPad Prism Version 5.01 (GraphPad Software, Inc.). The same software was used to calculate the half-maximal adhesion inhibitory concentration (IC_50_) of cilengitide. The results are presented as means ± SEM.

## SUPPLEMENTARY FIGURES AND TABLES




