# Supplementary material for: Targeting αvβ3 and αvβ5 integrins inhibits pulmonary metastasis in an intratibial xenograft osteosarcoma mouse model
Source: Oncotarget. 2016 Jul 7;7(34):55141–54. doi: 10.18632/oncotarget.10461 (PMC5342407; doi:10.18632/oncotarget.10461)
Supplement: Supplementary file 1 [file oncotarget-07-55141-s001.pdf]

Targeting αvβ3 and αvβ5 integrins inhibits pulmonary metastasis in an intratibial xenograft osteosarcoma mouse model

SUPPLEMENTARY FIGURES AND TABLES

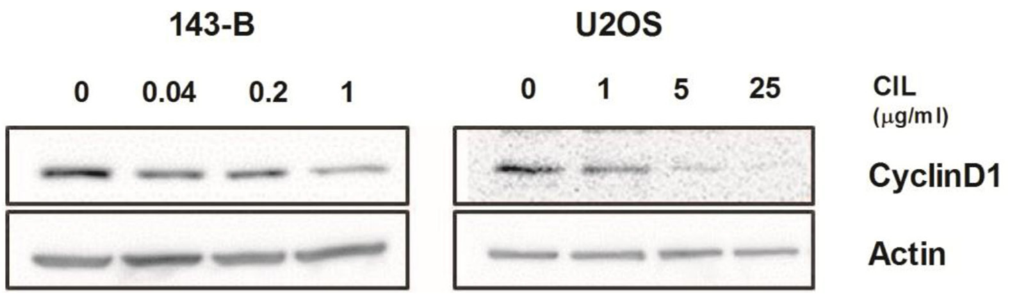

Supplementary Figure S1: Effects of cilengitide on CyclinD1 expression. 143-B and U2OS cells were incubated for 24 hours in the absence or presence of cilengitide (CIL) at indicated concentrations. The cell lysates were analyzed on Western blots, using the indicated antibodies.

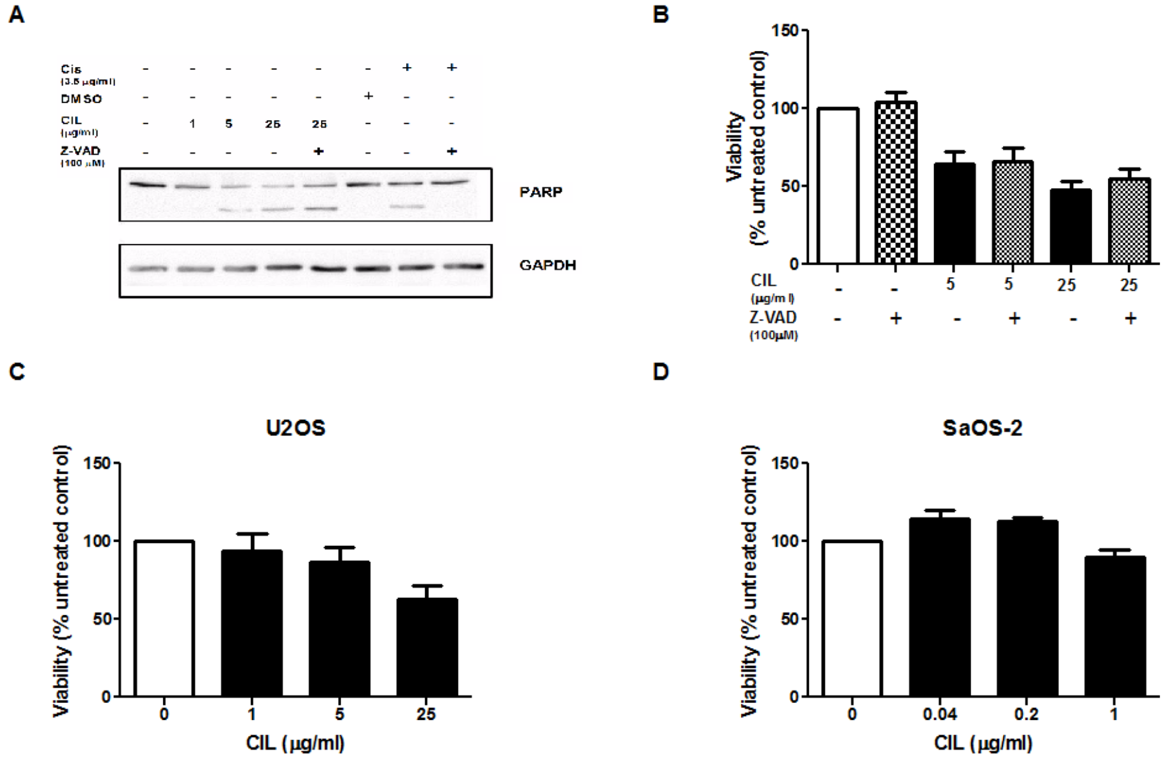

Supplementary Figure S2: Effects of cilengitide on PARP cleavage and cell viability in osteosarcoma cell lines. A. U2OS cells were left untreated or were treated with Z-VAD for 2 hours prior to 24 hours incubation with indicated concentrations of CIL or of cisplatin (CIS) or DMSO as controls. The cell lysates were analyzed on Western blots with antibodies indicated on the right (representative experiment carried out 3 times). **B.** Viability, assessed in a WST-1 assay, of U2OS cells pre-incubated for 2 hours in the absence or presence of the pan caspase inhibitor Z-VAD before treatment for 24 hours with indicated concentrations of CIL. **C.** and **D.** Viability, assessed in WST-1 assays, in indicated osteosarcoma cell lines treated for 6 hours with indicated concentrations of CIL. Values in B, C and D are the mean ± SEM of three independent experiments.

Supplementary Table S1: αvβ3 and αvβ5 integrin expression examined by tissue-microarray based immunohistochemistry in tumor tissues collected from osteosarcoma patients. BX, biopsy; RX, resection; RZ, recurrence; META (L), lung metastases; META (B), bone metastases; META (ST), soft tissue metastases. The immunohistochemical evaluation of αvβ3 integrin expression included the examination of the staining pattern and was carried out individually for tumor and stromal cells. The absence (negative) or presence (positive) of immunostaining was judged by eye. Grading of αvβ5 integrin immunostaining, based on the intensity and the percentage of immunostained area, was done with a custom made MATLAB (v2009b, Mathworks Inc) program as described (43). Exclusion criteria included damaged TMA spots or a complete absence of tumor cells.

See Supplementary File 1

Supplementary Table S2: Clinical characteristics of osteosarcoma patients

**Gender**	**n=86**	**%**
Male	53	61.6
Female	33	38.4
**Age**	**n=86**	**%**
<10	11	12.8
10 to 19	37	43.0
20 to 29	18	20.9
30 to 39	7	8.1
40 to 49	8	9.3
50 to 59	3	3.5
60 to 69	2	2.3
**Tumor type**	**n=86**	**%**
Osteoblastic	56	65.1
Chondroblastic	15	17.4
Fibroblastic	10	11.6
Teleangiectatic	5	5.8
**Anatomic site**	**n=86**	**%**
Extremities	66	76.7
Spine and pelvis	14	16.3
Face	6	7.0
**Chemotherapy response**	**n=64**	**%**
Responders (S-K-I-III)	35	54.7
Non-responders (S-K-IV-VI)	29	45.3
**Metastasis**	**n=86**	**%**
No metastasis	40	46.5
Total metastasis	46	53.5

Supplementary Table S3: Primer pairs used in the RT-PCR analysis

Gene Name (human)	Forward and Reverse Primer Sequence
CTGF	Fwd: CGAGCCCAAGGACCAAA<br>Rev: TCCCACAGGTCTTGGAACA
CYR61	Fwd: CCAATGACAACCCTGAGT<br>Rev: CTCAAACATCCAGCGTAAGT
ANKRD1	Fwd: CACTTCTAGCCCACCCTGTGA<br>Rev: CCACAGGTTCCGTAATGATTT
GAPDH	Fwd: AAGGCTGGGGCTCATTTGCAGG<br>Rev: AGTTGGTGGTGCAGGAGGCA
