# Supplementary material for: Targeting αvβ3 and αvβ5 integrins inhibits pulmonary metastasis in an intratibial xenograft osteosarcoma mouse model
Source: Oncotarget. 2016 Jul 7;7(34):55141–54. doi: 10.18632/oncotarget.10461 (PMC5342407; doi:10.18632/oncotarget.10461)
Supplement: Supplementary file 2 [file oncotarget-07-55141-s002.docx]

**Supplementary Table S1. αvβ3 and αvβ5 integrin expression examined by tissue-microarray based immunohistochemistry in tumor tissues collected from osteosarcoma patients.** BX, biopsy; RX, resection; RZ, recurrence; META (L), lung metastases; META (B), bone metastases; META (ST), soft tissue metastases. The immunohistochemical evaluation of αvβ3 integrin expression included the examination of the staining pattern and was carried out individually for tumor and stromal cells. The absence (negative) or presence (positive) of immunostaining was judged by eye. Grading of αvβ5 integrin immunostaining, based on the intensity and the percentage of immunostained area, was done with a custom made MATLAB (v2009b, Mathworks Inc) program as described (43). Exclusion criteria included damaged TMA spots or a complete absence of tumor cells.

| Tissue Type | Patient Number | Spot Number | αvβ3 Staining Pattern: 0=none, 1=tumor cells, 2=stroma, 3=both | αvβ3 Intensity Score: 0=excluded, 1=negative, 2=positive | αvβ5 Grading Score: 0=excluded,  1= negative,  2,3= positive |
| --- | --- | --- | --- | --- | --- |
| BX | 1 | 1 | 0 | 0 | 1 |
| BX | 1 | 2 | 2 | 2 | 0 |
| BX | 1 | 3 | 2 | 2 | 3 |
| BX | 1 | 4 | 0 | 0 | 0 |
| RX | 1 | 5 | 0 | 1 | 1 |
| RX | 1 | 6 | 0 | 1 | 1 |
| RZ | 1 | 7 | 0 | 1 | 1 |
| RZ | 1 | 8 | 0 | 1 | 2 |
| RZ | 1 | 9 | 0 | 1 | 2 |
| RZ | 1 | 10 | 0 | 1 | 2 |
| RZ | 1 | 11 | 0 | 1 | 2 |
| RZ | 1 | 12 | 0 | 1 | 3 |
| RZ | 1 | 13 | 2 | 2 | 2 |
| RZ | 1 | 14 | 2 | 2 | 2 |
| META(L) | 1 | 15 | 0 | 1 | 1 |
| META(L) | 1 | 16 | 0 | 1 | 1 |
| BX | 2 | 17 | 2 | 2 | 2 |
| BX | 2 | 18 | 0 | 0 | 0 |
| BX | 2 | 19 | 0 | 1 | 2 |
| BX | 2 | 20 | 0 | 1 | 1 |
| BX | 3 | 21 | 0 | 0 | 0 |
| BX | 3 | 22 | 0 | 1 | 2 |
| BX | 3 | 23 | 0 | 0 | 2 |
| BX | 3 | 24 | 0 | 0 | 3 |
| META(B) | 3 | 25 | 2 | 2 | 2 |
| META(B) | 3 | 26 | 2 | 2 | 2 |
| BX | 4 | 27 | 2 | 2 | 3 |
| BX | 4 | 28 | 2 | 2 | 2 |
| RX | 4 | 29 | 2 | 2 | 0 |
| RX | 4 | 30 | 0 | 0 | 0 |
| BX | 5 | 31 | 0 | 1 | 3 |
| BX | 5 | 32 | 2 | 2 | 3 |
| RX | 5 | 33 | 2 | 2 | 1 |
| RX | 5 | 34 | 0 | 0 | 0 |
| RX | 6 | 35 | 0 | 0 | 0 |
| RX | 6 | 36 | 0 | 1 | 1 |
| META(L) | 7 | 37 | 2 | 2 | 2 |
| META(L) | 7 | 38 | 2 | 2 | 2 |
| META(L) | 7 | 39 | 2 | 2 | 3 |
| META(L) | 7 | 40 | 2 | 2 | 3 |
| META(L) | 7 | 41 | 3 | 2 | 1 |
| META(L) | 7 | 42 | 3 | 2 | 1 |
| META(L) | 7 | 43 | 2 | 2 | 3 |
| META(L) | 7 | 44 | 2 | 2 | 3 |
| META(L) | 7 | 45 | 2 | 2 | 3 |
| META(L) | 7 | 46 | 2 | 2 | 3 |
| META(L) | 7 | 47 | 2 | 2 | 1 |
| META(L) | 7 | 48 | 2 | 2 | 1 |
| META(ST) | 7 | 49 | 0 | 1 | 2 |
| META(ST) | 7 | 50 | 0 | 1 | 2 |
| META(L) | 8 | 51 | 2 | 2 | 3 |
| META(L) | 8 | 52 | 2 | 2 | 3 |
| META(L) | 8 | 53 | 2 | 2 | 3 |
| META(L) | 8 | 54 | 0 | 0 | 0 |
| BX | 10 | 55 | 0 | 0 | 0 |
| BX | 10 | 56 | 0 | 1 | 1 |
| RX | 10 | 57 | 2 | 2 | 0 |
| RX | 10 | 58 | 2 | 2 | 0 |
| RZ | 10 | 59 | 0 | 1 | 2 |
| RZ | 10 | 60 | 0 | 1 | 3 |
| RZ | 10 | 61 | 0 | 1 | 3 |
| RZ | 10 | 62 | 0 | 1 | 3 |
| BX | 11 | 63 | 2 | 2 | 3 |
| BX | 11 | 64 | 0 | 1 | 2 |
| RX | 11 | 65 | 0 | 1 | 1 |
| RX | 11 | 66 | 0 | 1 | 3 |
| RX | 12 | 67 | 0 | 1 | 0 |
| RX | 12 | 68 | 0 | 1 | 1 |
| RZ | 12 | 69 | 0 | 0 | 0 |
| RZ | 12 | 70 | 0 | 1 | 2 |
| BX | 13 | 71 | 2 | 2 | 1 |
| BX | 13 | 72 | 2 | 2 | 1 |
| RX | 13 | 73 | 2 | 2 | 2 |
| RX | 13 | 74 | 2 | 2 | 3 |
| RX | 14 | 75 | 0 | 0 | 1 |
| RX | 14 | 76 | 0 | 0 | 1 |
| META(L) | 14 | 77 | 2 | 2 | 1 |
| META(L) | 14 | 78 | 2 | 2 | 1 |
| BX | 15 | 79 | 0 | 1 | 1 |
| BX | 15 | 80 | 0 | 1 | 2 |
| RX | 15 | 81 | 2 | 2 | 2 |
| RX | 15 | 82 | 0 | 1 | 1 |
| RX | 16 | 83 | 0 | 0 | 1 |
| RX | 16 | 84 | 0 | 0 | 1 |
| BX | 17 | 85 | 0 | 0 | 0 |
| BX | 17 | 86 | 2 | 2 | 2 |
| RX | 17 | 87 | 2 | 2 | 2 |
| RX | 17 | 88 | 0 | 0 | 0 |
| BX | 19 | 89 | 2 | 2 | 2 |
| BX | 19 | 90 | 2 | 2 | 3 |
| RX | 19 | 91 | 2 | 2 | 2 |
| RX | 19 | 92 | 2 | 2 | 2 |
| BX | 20 | 93 | 2 | 2 | 2 |
| BX | 20 | 94 | 2 | 2 | 2 |
| BX | 22 | 95 | 2 | 2 | 1 |
| BX | 22 | 96 | 2 | 2 | 2 |
| RX | 22 | 97 | 2 | 2 | 0 |
| RX | 22 | 98 | 2 | 2 | 0 |
| RX | 23 | 99 | 1 | 2 | 1 |
| RX | 23 | 100 | 0 | 0 | 1 |
| RX | 24 | 101 | 2 | 2 | 2 |
| RX | 24 | 102 | 2 | 2 | 1 |
| BX | 25 | 103 | 2 | 2 | 2 |
| BX | 25 | 104 | 0 | 0 | 0 |
| RX | 25 | 105 | 0 | 1 | 1 |
| RX | 25 | 106 | 0 | 0 | 0 |
| RX | 26 | 107 | 0 | 1 | 3 |
| RX | 26 | 108 | 2 | 2 | 2 |
| BX | 27 | 109 | 0 | 0 | 0 |
| BX | 27 | 110 | 0 | 0 | 0 |
| RX | 27 | 111 | 0 | 1 | 3 |
| RX | 27 | 112 | 0 | 1 | 3 |
| RX | 28 | 113 | 2 | 2 | 1 |
| RX | 28 | 114 | 2 | 2 | 1 |
| BX | 29 | 115 | 0 | 0 | 0 |
| BX | 29 | 116 | 0 | 0 | 0 |
| RX | 29 | 117 | 0 | 0 | 3 |
| RX | 29 | 118 | 0 | 1 | 3 |
| RX | 29 | 119 | 2 | 2 | 3 |
| RX | 29 | 120 | 2 | 2 | 3 |
| RX | 29 | 121 | 2 | 2 | 2 |
| RX | 29 | 122 | 2 | 2 | 3 |
| BX | 30 | 123 | 2 | 2 | 1 |
| BX | 30 | 124 | 2 | 2 | 2 |
| RX | 31 | 125 | 2 | 2 | 1 |
| RX | 31 | 126 | 2 | 2 | 1 |
| BX | 32 | 127 | 0 | 0 | 1 |
| BX | 32 | 128 | 2 | 2 | 2 |
| BX | 33 | 129 | 2 | 2 | 2 |
| BX | 33 | 130 | 2 | 2 | 2 |
| BX | 34 | 131 | 0 | 1 | 0 |
| BX | 34 | 132 | 0 | 1 | 0 |
| RX | 34 | 133 | 1 | 2 | 1 |
| RX | 34 | 134 | 0 | 0 | 0 |
| RZ | 34 | 135 | 2 | 2 | 2 |
| RZ | 34 | 136 | 2 | 2 | 1 |
| BX | 35 | 137 | 2 | 2 | 3 |
| BX | 35 | 138 | 0 | 0 | 0 |
| BX | 36 | 139 | 2 | 2 | 1 |
| BX | 36 | 140 | 2 | 2 | 1 |
| RX | 37 | 141 | 0 | 0 | 2 |
| RX | 37 | 142 | 0 | 1 | 2 |
| RX | 37 | 143 | 0 | 1 | 2 |
| RX | 37 | 144 | 0 | 1 | 1 |
| BX | 38 | 145 | 0 | 1 | 3 |
| BX | 38 | 146 | 0 | 1 | 3 |
| BX | 39 | 147 | 2 | 2 | 3 |
| BX | 39 | 148 | 2 | 2 | 3 |
| RX | 39 | 149 | 2 | 2 | 2 |
| RX | 39 | 150 | 0 | 0 | 0 |
| RX | 39 | 151 | 0 | 1 | 1 |
| RX | 39 | 152 | 2 | 2 | 1 |
| BX | 40 | 153 | 2 | 2 | 3 |
| BX | 40 | 154 | 2 | 2 | 3 |
| RX | 40 | 155 | 1 | 2 | 1 |
| RX | 40 | 156 | 1 | 2 | 1 |
| RX | 40 | 157 | 1 | 2 | 1 |
| RX | 40 | 158 | 1 | 2 | 1 |
| META(L) | 40 | 159 | 2 | 2 | 3 |
| META(L) | 40 | 160 | 2 | 2 | 3 |
| META(L) | 41 | 161 | 0 | 1 | 1 |
| META(L) | 41 | 162 | 0 | 1 | 2 |
| META(L) | 41 | 163 | 0 | 1 | 1 |
| META(L) | 41 | 164 | 0 | 1 | 1 |
| META(L) | 41 | 165 | 2 | 2 | 3 |
| META(L) | 41 | 166 | 2 | 2 | 2 |
| BX | 42 | 167 | 2 | 2 | 3 |
| BX | 42 | 168 | 2 | 2 | 3 |
| RX | 42 | 169 | 1 | 2 | 1 |
| RX | 42 | 170 | 1 | 2 | 2 |
| BX | 43 | 171 | 2 | 0 | 3 |
| BX | 43 | 172 | 0 | 0 | 3 |
| RX | 43 | 173 | 2 | 2 | 3 |
| RX | 43 | 174 | 2 | 2 | 2 |
| RX | 44 | 175 | 0 | 1 | 2 |
| RX | 44 | 176 | 0 | 0 | 0 |
| RX | 44 | 177 | 3 | 2 | 1 |
| RX | 44 | 178 | 2 | 2 | 2 |
| BX | 45 | 179 | 1 | 2 | 2 |
| BX | 45 | 180 | 1 | 2 | 2 |
| RX | 45 | 181 | 1 | 2 | 2 |
| RX | 45 | 182 | 1 | 2 | 2 |
| RX | 46 | 183 | 2 | 2 | 1 |
| RX | 46 | 184 | 2 | 2 | 1 |
| RX | 46 | 185 | 2 | 2 | 1 |
| RX | 46 | 186 | 1 | 2 | 1 |
| BX | 47 | 187 | 0 | 1 | 3 |
| BX | 47 | 188 | 0 | 1 | 3 |
| RX | 47 | 189 | 1 | 2 | 2 |
| RX | 47 | 190 | 1 | 2 | 2 |
| BX | 48 | 191 | 2 | 2 | 3 |
| BX | 48 | 192 | 2 | 2 | 2 |
| RX | 48 | 193 | 1 | 2 | 2 |
| RX | 48 | 194 | 1 | 2 | 3 |
| META(L) | 48 | 195 | 0 | 0 | 3 |
| META(L) | 48 | 196 | 0 | 0 | 3 |
| META(L) | 48 | 197 | 2 | 2 | 3 |
| META(L) | 48 | 198 | 2 | 2 | 3 |
| META(L) | 48 | 199 | 2 | 2 | 3 |
| META(L) | 48 | 200 | 2 | 2 | 3 |
| RX | 49 | 201 | 0 | 1 | 1 |
| RX | 49 | 202 | 0 | 1 | 1 |
| BX | 50 | 203 | 0 | 0 | 3 |
| BX | 50 | 204 | 2 | 2 | 2 |
| BX | 50 | 205 | 0 | 0 | 0 |
| BX | 50 | 206 | 2 | 2 | 2 |
| RX | 50 | 207 | 0 | 1 | 2 |
| RX | 50 | 208 | 0 | 1 | 1 |
| META(L) | 51 | 209 | 0 | 0 | 3 |
| META(L) | 51 | 210 | 0 | 0 | 0 |
| META(L) | 51 | 211 | 2 | 2 | 3 |
| META(L) | 51 | 212 | 2 | 2 | 3 |
| BX | 52 | 213 | 0 | 1 | 1 |
| BX | 52 | 214 | 0 | 1 | 1 |
| RX | 52 | 215 | 0 | 1 | 2 |
| RX | 52 | 216 | 2 | 2 | 1 |
| Bx | 53 | 217 | 2 | 2 | 2 |
| BX | 53 | 218 | 0 | 1 | 2 |
| RX | 53 | 219 | 0 | 1 | 1 |
| RX | 53 | 220 | 0 | 0 | 0 |
| BX | 54 | 221 | 0 | 1 | 1 |
| BX | 54 | 222 | 0 | 1 | 1 |
| RX | 54 | 223 | 2 | 2 | 1 |
| RX | 54 | 224 | 2 | 2 | 1 |
| META(B) | 54 | 225 | 0 | 0 | 0 |
| META(B) | 54 | 226 | 0 | 0 | 0 |
| BX | 55 | 227 | 2 | 2 | 2 |
| BX | 55 | 228 | 2 | 2 | 3 |
| RX | 55 | 229 | 3 | 2 | 1 |
| RX | 55 | 230 | 3 | 2 | 0 |
| RX | 55 | 231 | 1 | 2 | 1 |
| RX | 55 | 232 | 1 | 2 | 1 |
| BX | 56 | 233 | 2 | 2 | 2 |
| BX | 56 | 234 | 0 | 0 | 3 |
| RX | 56 | 235 | 1 | 2 | 1 |
| RX | 56 | 236 | 1 | 2 | 1 |
| RX | 56 | 237 | 1 | 2 | 1 |
| RX | 56 | 238 | 1 | 2 | 1 |
| BX | 57 | 239 | 0 | 1 | 1 |
| BX | 57 | 240 | 0 | 1 | 1 |
| RX | 57 | 241 | 2 | 2 | 1 |
| RX | 57 | 242 | 0 | 1 | 1 |
| RZ | 57 | 243 | 0 | 1 | 3 |
| RZ | 57 | 244 | 0 | 1 | 3 |
| META(L) | 57 | 245 | 2 | 2 | 3 |
| META(L) | 57 | 246 | 2 | 2 | 3 |
| META(L) | 57 | 247 | 0 | 1 | 1 |
| META(L) | 57 | 248 | 0 | 1 | 1 |
| META(B) | 57 | 249 | 0 | 1 | 3 |
| META(B) | 57 | 250 | 2 | 2 | 3 |
| BX | 58 | 251 | 0 | 1 | 1 |
| BX | 58 | 252 | 0 | 1 | 1 |
| BX | 58 | 253 | 2 | 2 | 2 |
| BX | 58 | 254 | 2 | 2 | 1 |
| BX | 59 | 255 | 2 | 2 | 2 |
| BX | 59 | 256 | 0 | 1 | 1 |
| RX | 59 | 257 | 2 | 2 | 1 |
| RX | 59 | 258 | 2 | 2 | 1 |
| BX | 60 | 259 | 0 | 1 | 3 |
| BX | 60 | 260 | 0 | 1 | 3 |
| RX | 60 | 261 | 2 | 2 | 1 |
| RX | 60 | 262 | 0 | 0 | 1 |
| BX | 61 | 263 | 2 | 2 | 3 |
| BX | 61 | 264 | 2 | 2 | 3 |
| RX | 61 | 265 | 2 | 2 | 1 |
| RX | 61 | 266 | 0 | 0 | 0 |
| RX | 61 | 267 | 2 | 2 | 1 |
| RX | 61 | 268 | 2 | 2 | 1 |
| BX | 62 | 269 | 2 | 2 | 3 |
| BX | 62 | 270 | 0 | 0 | 0 |
| RX | 62 | 271 | 2 | 2 | 1 |
| RX | 62 | 272 | 2 | 2 | 1 |
| META(L) | 62 | 273 | 2 | 2 | 2 |
| META(L) | 62 | 274 | 2 | 2 | 1 |
| RX | 63 | 275 | 2 | 2 | 1 |
| RX | 63 | 276 | 2 | 2 | 1 |
| RX | 63 | 277 | 2 | 2 | 1 |
| RX | 63 | 278 | 2 | 2 | 1 |
| RZ | 63 | 279 | 0 | 0 | 0 |
| RZ | 63 | 280 | 0 | 0 | 0 |
| BX | 64 | 281 | 2 | 2 | 3 |
| BX | 64 | 282 | 2 | 2 | 3 |
| RX | 64 | 283 | 2 | 2 | 1 |
| RX | 64 | 284 | 2 | 2 | 1 |
| BX | 65 | 285 | 2 | 2 | 3 |
| BX | 65 | 286 | 2 | 2 | 2 |
| BX | 65 | 287 | 0 | 0 | 3 |
| BX | 65 | 288 | 2 | 2 | 3 |
| RX | 65 | 289 | 2 | 2 | 1 |
| RX | 65 | 290 | 0 | 1 | 1 |
| BX | 66 | 291 | 2 | 2 | 3 |
| BX | 66 | 292 | 2 | 2 | 3 |
| RX | 66 | 293 | 2 | 2 | 1 |
| RX | 66 | 294 | 2 | 2 | 1 |
| RX | 66 | 295 | 0 | 1 | 2 |
| RX | 66 | 296 | 0 | 1 | 2 |
| BX | 67 | 297 | 0 | 1 | 2 |
| BX | 67 | 298 | 2 | 2 | 2 |
| META(B) | 67 | 299 | 2 | 2 | 2 |
| META(B) | 67 | 300 | 2 | 2 | 2 |
| BX | 68 | 301 | 0 | 0 | 0 |
| BX | 68 | 302 | 0 | 0 | 0 |
| RX | 68 | 303 | 0 | 1 | 1 |
| RX | 68 | 304 | 2 | 2 | 1 |
| RX | 68 | 305 | 2 | 2 | 1 |
| RX | 68 | 306 | 2 | 2 | 1 |
| META(L) | 68 | 307 | 0 | 1 | 0 |
| META(L) | 68 | 308 | 0 | 0 | 2 |
| BX | 69 | 309 | 2 | 2 | 3 |
| BX | 69 | 310 | 2 | 2 | 2 |
| RX | 69 | 311 | 2 | 2 | 3 |
| RX | 69 | 312 | 2 | 2 | 1 |
| BX | 70 | 313 | 0 | 0 | 0 |
| BX | 70 | 314 | 0 | 1 | 1 |
| RX | 71 | 315 | 2 | 2 | 0 |
| RX | 71 | 316 | 0 | 1 | 1 |
| BX | 72 | 317 | 0 | 1 | 2 |
| BX | 72 | 318 | 2 | 2 | 2 |
| RX | 72 | 319 | 0 | 1 | 1 |
| RX | 72 | 320 | 0 | 1 | 2 |
| RX | 72 | 321 | 0 | 1 | 3 |
| RX | 72 | 322 | 2 | 2 | 3 |
| RZ | 72 | 323 | 0 | 1 | 3 |
| RZ | 72 | 324 | 2 | 2 | 3 |
| RZ | 72 | 325 | 2 | 2 | 2 |
| RZ | 72 | 326 | 0 | 1 | 3 |
| RX | 73 | 327 | 2 | 2 | 2 |
| RX | 73 | 328 | 2 | 2 | 3 |
| BX | 74 | 329 | 0 | 1 | 2 |
| BX | 74 | 330 | 0 | 1 | 2 |
| RX | 74 | 331 | 2 | 2 | 2 |
| RX | 74 | 332 | 0 | 0 | 1 |
| BX | 75 | 333 | 0 | 1 | 1 |
| BX | 75 | 334 | 2 | 2 | 1 |
| RX | 75 | 335 | 2 | 2 | 3 |
| RX | 75 | 336 | 2 | 2 | 1 |
| RX | 75 | 337 | 2 | 2 | 2 |
| RX | 75 | 338 | 2 | 2 | 3 |
| BX | 76 | 339 | 0 | 1 | 2 |
| BX | 76 | 340 | 0 | 0 | 2 |
| RX | 76 | 341 | 0 | 1 | 1 |
| RX | 76 | 342 | 0 | 1 | 2 |
| BX | 77 | 343 | 2 | 2 | 2 |
| BX | 77 | 344 | 2 | 2 | 0 |
| RX | 77 | 345 | 2 | 2 | 1 |
| RX | 77 | 346 | 0 | 1 | 1 |
| BX | 78 | 347 | 2 | 2 | 3 |
| BX | 78 | 348 | 2 | 2 | 2 |
| RX | 78 | 349 | 2 | 2 | 1 |
| RX | 78 | 350 | 2 | 2 | 1 |
| RX | 78 | 351 | 2 | 2 | 1 |
| RX | 78 | 352 | 0 | 1 | 0 |
| BX | 79 | 353 | 2 | 2 | 3 |
| BX | 79 | 354 | 2 | 2 | 3 |
| RX | 79 | 355 | 0 | 1 | 1 |
| RX | 79 | 356 | 2 | 2 | 1 |
| BX | 80 | 357 | 2 | 2 | 3 |
| BX | 80 | 358 | 2 | 2 | 2 |
| RX | 80 | 359 | 2 | 2 | 1 |
| RX | 80 | 360 | 2 | 2 | 0 |
| RX | 81 | 361 | 2 | 2 | 1 |
| RX | 81 | 362 | 0 | 1 | 1 |
| RX | 81 | 363 | 2 | 2 | 1 |
| RX | 81 | 364 | 0 | 1 | 1 |
| BX | 82 | 365 | 0 | 0 | 0 |
| BX | 82 | 366 | 2 | 2 | 3 |
| RX | 82 | 367 | 2 | 2 | 2 |
| RX | 82 | 368 | 2 | 2 | 2 |
| BX | 83 | 369 | 0 | 0 | 0 |
| BX | 83 | 370 | 2 | 2 | 3 |
| BX | 83 | 371 | 2 | 2 | 2 |
| BX | 83 | 372 | 0 | 0 | 0 |
| RX | 83 | 373 | 2 | 2 | 2 |
| RX | 83 | 374 | 2 | 2 | 2 |
| RX | 83 | 375 | 2 | 2 | 3 |
| RX | 83 | 376 | 2 | 2 | 2 |
| BX | 84 | 377 | 0 | 1 | 1 |
| BX | 84 | 378 | 2 | 2 | 2 |
| BX | 85 | 379 | 2 | 2 | 3 |
| BX | 85 | 380 | 0 | 0 | 0 |
| RX | 85 | 381 | 2 | 2 | 3 |
| RX | 85 | 382 | 2 | 2 | 3 |
| RX | 86 | 383 | 2 | 2 | 1 |
| RX | 86 | 384 | 2 | 2 | 1 |
| BX | 87 | 385 | 2 | 2 | 2 |
| BX | 87 | 386 | 2 | 2 | 3 |
| BX | 88 | 387 | 0 | 1 | 2 |
| BX | 88 | 388 | 2 | 2 | 2 |
| BX | 89 | 389 | 2 | 2 | 2 |
| BX | 89 | 390 | 0 | 0 | 0 |
| RX | 89 | 391 | 2 | 2 | 1 |
| RX | 89 | 392 | 0 | 0 | 0 |
| RX | 89 | 393 | 0 | 0 | 0 |
| RX | 89 | 394 | 0 | 0 | 1 |
| RX | 89 | 395 | 2 | 2 | 2 |
| RX | 89 | 396 | 2 | 2 | 3 |
| BX | 90 | 397 | 1 | 2 | 1 |
| BX | 90 | 398 | 1 | 2 | 1 |
| BX | 91 | 399 | 1 | 2 | 1 |
| BX | 91 | 400 | 1 | 2 | 1 |
| BX | 93 | 401 | 2 | 2 | 3 |
| BX | 93 | 402 | 2 | 2 | 2 |
| BX | 94 | 403 | 2 | 2 | 1 |
| BX | 94 | 404 | 2 | 2 | 1 |
